# Knowledge and Attitude of Midwifery Students on Oral Health Care

**DOI:** 10.3390/dj7030083

**Published:** 2019-08-01

**Authors:** Sepideh Seyedzadeh Sabounchi, Shabnam Seyedzadeh Sabounchi, Maryam Safari

**Affiliations:** 1 Prosthodontics Department, College of Dentistry, New York University, New York, NY 10010, USA; 2 College of Community and Public Affairs, Binghamton University, New York, NY 13902, USA; 3 Private Practice, Tehran 19839, Iran

**Keywords:** educational intervention, midwifery, student, pregnancy, women, dental, oral health

## Abstract

Midwifery students can have an important role in transferring oral health care information to expecting mothers. The aim of this study was to assess the effect of an educational intervention on knowledge and attitudes of midwifery students on oral health in pregnancy. Study population consisted of 60 midwifery students in a Midwifery School in Iran who were randomly allocated into case and control groups. Self-administered questionnaires were distributed before, immediately after the intervention and also three months later. The validity and reliability of the questionnaire was confirmed at the beginning. Mean total pre-test knowledge scores from total 10 in the interventional and control groups were 4.63 ± 0.25 (Standard Error, SE) and 4.79 ± 0.31 (SE) respectively. After three months scores reached to 8.87 ± 0.15 (SE) in the interventional and 5.57 ± 0.29 (SE) in the control groups. Mean attitude pre-test scores in the interventional group was 27.23 ± 0.75 (SE) and after the intervention reached to 31.13 ± 0.25 (SE). Lecture-based educational intervention improved the knowledge and attitudes of midwifery students on oral health care in pregnancy. Incorporating courses on oral health in pregnancy into the curricula of midwifery programs can be effective in promoting oral health care in pregnant women.

## 1. Introduction

Oral health is considered an important component of general health and poor oral health can have an adverse effect on the quality of life [1]. Oral health is especially critical during pregnancy since pregnant women undergo physiologic changes such as hormonal alterations, diet modification and concurrent vomiting and acidity in the mouth which can increase dental decay rates. Dental decay prevalence in pregnancy has been recorded as 74% in previous convenience samples in Thailand and 99.9% in Lithuania [2,3]. Pregnant women’s decreased interest in oral hygienic behaviors is another factor contributing to the development of tooth decay, gum and periodontal diseases [4]. Hormonal changes in pregnancy influence gingival health and are responsible for highly intensified inflammation reactions such as gingivitis (gingival inflammation). However, there is a wide range, from 30 to 100 percent variance in the rates of gingivitis during pregnancy [2,5,6]. In pregnancy, adjustments in estrogen and progesterone hormones can lead to more inflammatory reactions and increase permeability of capillaries and collagen formation [7]. Saliva composition also changes during pregnancy such that calcium and phosphate ions are reduced leading to pH reduction, which together provide a better environment for bacterial aggregation in the gums [8].

Studies have demonstrated an association between gingival or periodontal diseases in pregnant mothers and undesirable prenatal outcomes including preeclampsia, premature birth, low birth weight and lengthy hospitalization at neonatal intensive care unit [8,9]. The existing literature on the association of periodontal disease with premature birth highlight the need for further investigations [10]. Adherence of mothers to oral health behaviors during pregnancy can significantly affect their own oral and dental health and consequently their babies.

Dental caries is an infectious disease caused by pathogenic agents which can be transferred to healthy teeth. Oral cariogenic bacteria can transfer from mother to baby through intimate physical contact and feeding habits and directly lead to development of early childhood dental decay in infants [10,11]. Children learn oral health behaviors by modeling their parents’ behavior [12]. Since mothers have a significant role in children’s training, mothers’ oral health habits can also influence the future baby’s oral health [13]. The prevalence of pregnant women visiting dentists during pregnancy was 30% to 31% in Iran, 33% in United Kingdom and 50% in Australia [14,15,16,17]. The main reasons for seeking care from a dentist by pregnant women in Iran were teeth extraction (36%) and tooth pain 27% [17]. A study on Iranian pregnant women in need of periodontal treatment found that they were more likely to deliver low birth weight babies than those without periodontal problems [18]. Promoting oral health care during pregnancy is recognized as a critical issue. 

One opportunity to improve the oral health care of pregnant women is during the care offered by gynecologists, obstetricians, midwives, and nurses [19]. As these groups of health providers are routinely visited, during and after the pregnancy, there is a promising opportunity during these visits to promote oral health of pregnant women [20]. Specifically, nurses and midwives can play a significant role as examiners of oral health and educators in pregnant women. In Iran’s health care system, midwives provide care to pregnant women and visit them regularly based on a national prenatal comprehensive program [21]. A study on Iranian pregnant women found that 73% had never been advised by their midwife to see a dentist [15]. Midwives can be educated on how to assess pregnant women’s oral health status and make referrals to dentists if necessary. Although currently there are some guidelines prepared by leading organizations involved in pregnant women’s health, there is no official educational course or program on oral health in the midwifery educational curricula in Iran, United States, and Australia [1,22,23].

Studies have shown limited involvement and deficiency in knowledge on oral health care during pregnancy among gynecologists-obstetricians, nurses, and midwives [15,19,24]. The aim of this study was to assess the effect of an educational intervention on midwifery students’ attitude and knowledge on oral health in pregnant women.

## 2. Materials and Methods 

This study was a true experimental interventional study with pre-test and post-test study design. Sixty students were selected randomly from the list of the second and third year bachelor midwifery students registered at a Medical Sciences University in west of Iran. The bachelor in midwifery is a four-year degree program. In Iran’s health care system nursing students and midwifery students have two different educational programs. Although they have to pass similar courses, but their programs and curricula are designed separately [23]. The inclusion criteria for participation in the study were being registered in the midwifery program of the university and not taken nursing courses. The students selected in the study did not have prior exposure to oral health care in pregnancy. 

Initial introduction to the workshop was provided to all students the day before the workshops were held. At this meeting students were assigned to two different intervention groups of case (type A) and control (type B), each consisted of 30 participants. Students were not aware which group they had been assigned. They were informed that this was a workshop on health care for midwifery students. It was explained to them that pre-test and post-test questionnaires would be distributed among them before, and immediately after the workshop, and three months later in the study. The data collected from the study participants had no identifiers such as name or address that could be traced to any of the students. The Ethics Committee of Hamadan University of Medical Sciences approved this study (IR.UMSHA.REC.1394.376) on 5 December 2015. 

All invited students after hearing about the study objectives, the risks, benefits and the voluntary nature of their participation, gave their consents orally. At this stage the pre-test questionnaires were distributed among all the participating students. They were requested to develop a code for themselves based on their birth date and the two first digit of student ID as a reference number. They were told to use the number on top of all three questionnaires that they were going to complete. In this way, the contents of the questionnaires completed by each participant could be compared according to the matched codes before and after the intervention. Also, the investigators were masked to the results by this coding method.

For both groups a 90-min lecture was provided by one of the authors M.S. The intervention group received lecture on oral health care in pregnancy, feeding habits, sugar intake, transmission of bacteria, oral hygiene, association of oral diseases with general health based on previous standardized educational content [1,25,26]. Also, basic oral health screening skills in pregnant women and babies were provided. Then they watched a 15-min video on oral hygiene behaviors and oral clinical examinations. The control group received a 90-min lecture on stress management in the school and hospital environment. The students in control group watched a 15-min video on stress management methods.

The questionnaire was consisted of three sections. The first section included background information on the age, past history of attendance to workshops, interest to attend workshops on “oral health” and eagerness to provide oral and dental health care for pregnant women. Interest and eagerness were evaluated on a five-point Likert scale and one represented not interested and five highly interested. Also, three additional questions were asked on current perceived knowledge on gingival health, dental decay and provision of oral health care to pregnant women. The details of knowledge and attitude questions in the three sections are summarized in Table 1.

The second section contained knowledge questions on three areas of gingival/periodontal diseases, dental caries and oral hygiene modalities. The questions were multiple choice with one correct answer. The correct answer was credited with one point if selected by the participant and zero if not selected. The third section was on attitude questions and responses were evaluated on a five-point Likert scale categorized by ordinal numbers from one to five. One represented as completely disagree and five was completely agree. The attitude questions were designed in both negative and positive directions in order to reduce predictability of the answers by the respondents.

**Figure 1:**
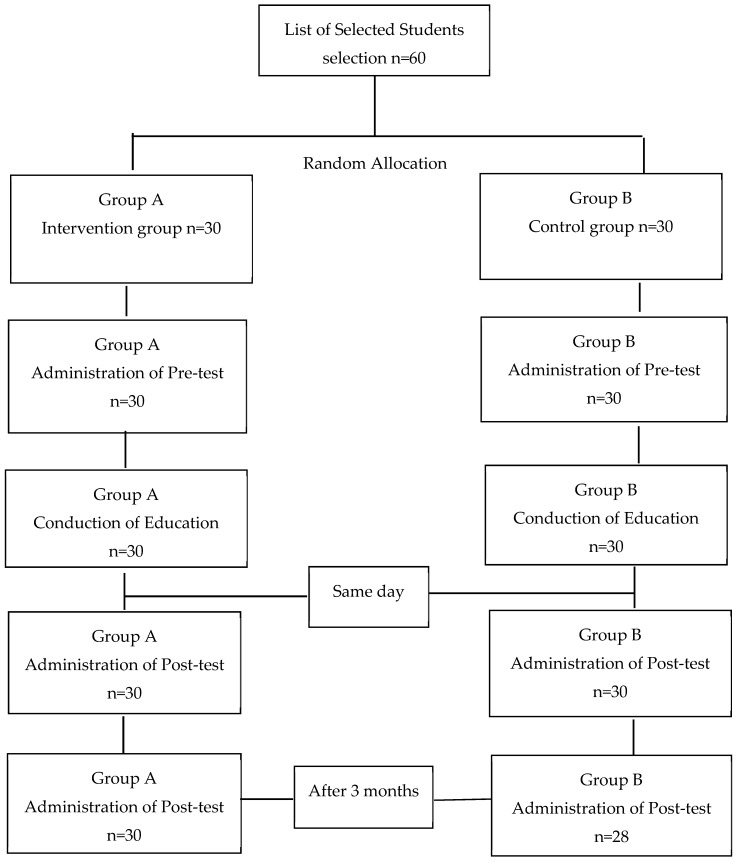
Flow chart of the interventional study.

At the end of each workshop, questionnaires were distributed again in the intervention and control groups. For determining the effect of time on the durability of the acquired knowledge, questionnaires were distributed after three months in both groups as well. The flow chart provides a detailed description of the steps taken in the study (Figure 1).

The content validity of the questionnaire was evaluated by determining the Content Validity Index (CVI) and Content Validity Ratio (CVR) [27,28]. Ten experts from the Pediatric, Oral Medicine, Periodontics and Community Oral Health Departments in Hamadan School of Dentistry were contacted, and they agreed to take part in the validation process of the questionnaire. CVR was computed for all items of the questionnaire by asking each of the 10 professionals to rate whether the knowledge or attitude construct measured by the question was “Essential”, “Useful but not essential”, or “Not necessary” [24]. CVI was measured by asking experts to score the relevancy, clarity and simplicity of each knowledge and attitude question using a four-point Likert-type scale. Score one represented lowest value and score four highest value. The CVI for each question was calculated through dividing the total number of high scored (3 or 4) questions by the total number of experts [27]. The collected opinions led to modifying six questions. The expert panel reached to a convergence in opinions and verified the revised questionnaire. 

The reliability of the questionnaire was calculated based on a pilot study on 30 midwifery first year students. Cronbach’s alpha coefficient was used to measure reliability for attitude which was 0.7. The reliability scores of equal or higher than 0.70 are considered satisfactory for a scale [29].

### Statistical Analysis

The SPSS software version 22 was used for statistical analysis. Pre-test and post-test knowledge and attitudes of participants in the intervention and control groups were described using frequency, mean and standard error of mean. Differences between the subgroups were assessed by Chi–square test and *T*-test. Analysis of Covariance (ANCOVA) was used to assess the effectiveness of the intervention on knowledge and attitudes of students involved in the study by controlling for pre-test knowledge and attitude. In case data did not meet the requirements of normality, Mann-Whitney U test was used to assess the between group difference. Association between different background variables and dependent variables was measured with Pearson’s correlation coefficients. Statistical significance was set to *p*-value < 0.05.

## 3. Results

In total sixty students participated in the study. The final number after three months follow-up in the control group was 28 since two students could not be reached and were excluded from the analysis. The average age of the participants was 22.33 ± 0.42 (SE) (Range 20 to 33 years) and 21.78 ± 0.65 (SE) (Range 17 to 35 years) in the intervention and control groups respectively and the differences were not statistically significant (*p* = 0.47). The analysis of the background section of the questionnaire showed that students’ mean interest on attending educational workshops on oral health care in pregnancy, was 4.1 ± 0.13 (SE) (Max = 5) in the intervention and 4.43 ± 0.13 (SE) (Max = 5) in the control groups. Their mean eagerness to provide oral health care to pregnant women was 3.9 ± 0.16 (SE) and 4.29 ± 0.13 (SE) in the intervention and control groups respectively. The differences among intervention and control groups on interest (*p* = 0.08) and eagerness (*p* = 0.07) were not statistically significant. 

**Figure 2:**
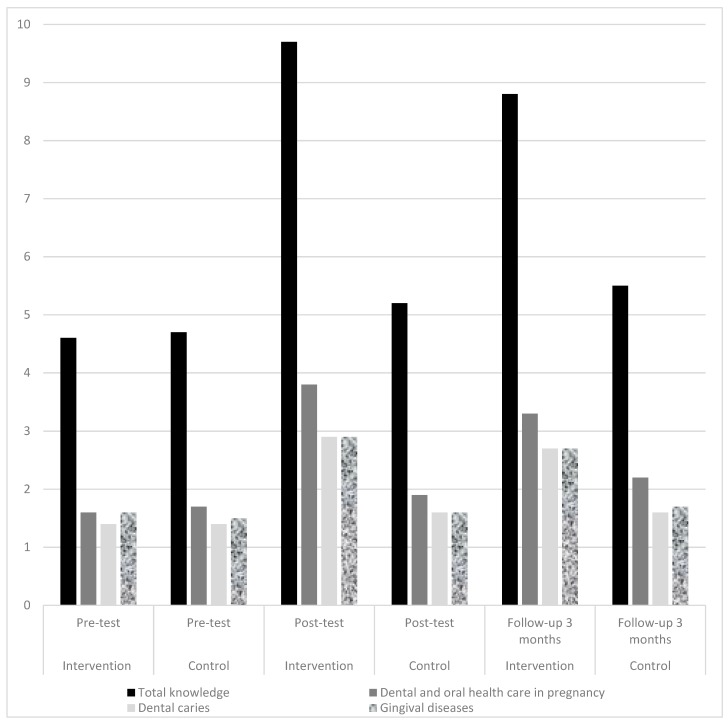
Mean knowledge scores in both intervention, and control groups at pre-test, post-test and follow-up 3 months.

In a one-way ANCOVA the effectiveness of the educational workshop whilst controlling for pre-test scores was examined. There was a significant difference in mean knowledge (*F*(1,55) = 390.06, p = 0.001) between the intervention and control groups after the intervention (Table 2). According to the partial Eta Squared of 0.87 and Cohen’s guidelines, the effect size is large and 87% of variance in post-test scores is explained by belonging to the intervention or control groups [30]. After three months the ANCOVA test showed that the intervention group had higher mean actual knowledge scores (8.87) compared to the control group (5.57) and the differences were also significant (*F*(1,55) = 146.16, *p* = 0.001). The changes in mean knowledge scores in pre-test, post-test and three months follow-ups are depicted in Table 2 and Figure 2.

**Figure 3:**
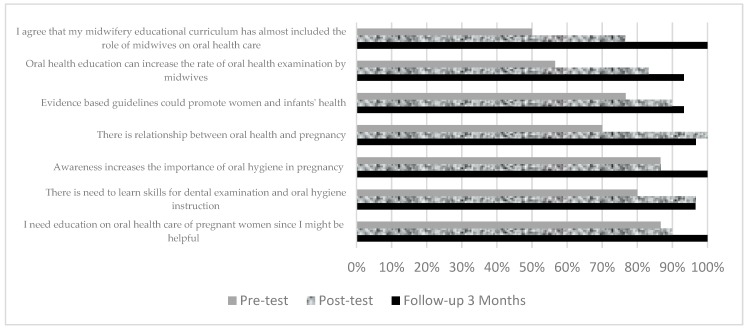
Comparison of positive attitude scores in the intervention group between pre-test, post-test, and follow-up 3 months.

The mean pre-test knowledge score in the intervention group was 4.63 ± 0.25 (SE) (Min = 0, Max = 10). After three months of intervention the knowledge score in this group reached to 8.87 ± 0.86 (SE) which was statistically higher than the control group (*p* ≤ 0.05). The mean attitude score in the intervention group was 27.23 ± 0.75 (SE) in the beginning and increased to 31.13 ± 0.25 (SE) after 3 months of intervention. The highest number of positive responses to attitude questions at pre-test in the intervention group was on the need to learn the skills for oral health care in pregnant women (86.7%) (Figure 3). 

**Figure 4:**
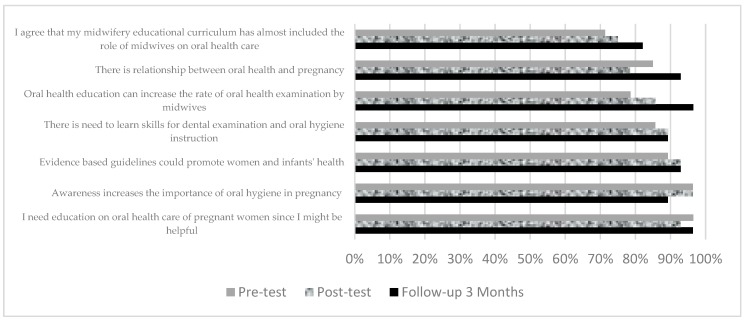
Comparison of positive attitude scores in the control group between pre-test, post-test, and follow-up 3 months.

In the control group the most positive agreement at pre-test was also on the need to learn the skills (96.5%) and the statement that awareness increases the importance of oral hygiene in pregnancy (96.4%) (Figure 4). 

Both the intervention and control groups showed the lowest agreement on the statement that in their opinion midwifery educational program has almost included the role of midwives on oral health care; which were 50% and 71.4% respectively. Results of ANCOVA analysis showed that the mean post-test attitude scores were significantly higher in the intervention group 31.41 compared to the control group 30.30 (*F*(1,55) = 2.18, *p* = 0.003). The effect size was 0.04 and in the low category [26]. However, after three months differences in the attitudes at pre-test and three months follow-up were not statistically significant among intervention and control groups (*p* = 0.05).

In Table 3 the pre-test, post-test and three months follow-up spearman correlation coefficients are shown. The numbers depict the correlation between total attitudes and actual knowledge with the interest to attend workshop and eagerness for provision of oral health care in pregnancy. The results showed significant direct relationships between total attitudes, and interest (*r* = 0.57) and eagerness (*r* = 0.48) in the intervention group at pre-test time interval (*p* ≤ 0.05). Total attitudes and interest to attend workshop were directly correlated in the intervention group at post-test interval as well (*r* = 0.43, *p* ≤ 0.05) (Table 3).

## 4. Discussion

The results of our study showed that three months after receiving the educational workshop on oral health in pregnancy, the knowledge and attitude of the midwifery students improved. The intervention group’s mean pre-test knowledge score was 4.6 from the total score of 10 and regarded low. In other studies, the mean knowledge score of midwifery students were recorded as 26.7 from total score of 100 [31], and 5.2 from eight [32], which were also undesirable. These results are also comparable to similar studies on gynecologists and their low mean knowledge score of 7.99 from the total score of 11 [33].

Attitude acts as a predisposition variable to the ability to provide oral health care examinations and care to pregnant women and eventually it can influence the midwives’ performance. Our study found that the intervention significantly improved students’ attitudes. Three areas of academic learning behavior, cognitive (knowledge), affective (attitude), and psychomotor (performance) domains are in bilateral associations with each other and there is possibility to predict one from the other [34,35]. However, there is an intermediate factor or willingness which may affect attitudes and actual performance [36]. Our study findings identified a statistically significant direct positive association between midwives’ attitudes and willingness to provide health care to pregnant women. 

Only 40% of the midwifery students in our study were aware how pregnancy hormonal imbalances can induce gingival diseases. Also, 33% believed that periodic dental check-ups for pregnant women are needed. Furthermore, nurse practitioners and certified nurse midwives delivering care to pregnant women had limited knowledge on periodontal disease [37]. Nurses believe that nursing school’s curricula on oral health care are insufficient [37]. Nurses are rarely delivering oral health examinations, referring patients to dentists and educating them on oral hygiene behaviors [38] According to the literature review midwifery and nurse practitioners’ knowledge on gingival disease knowledge is lower than gynecologists and obstetricians [20,39,40,41,42]. These results suggest that the importance of gingival diseases and their association with pregnancy outcomes are overlooked in the midwifery educational curriculum compared to the gynecology and obstetrician programs.

The knowledge of midwifery students in our study on referral to dentist was low. Referral rates for routine dental check-ups are low among gynecologists and obstetricians as well [39,40,43]. On the other hand, studies report a higher percentage of referral to dentists by medical practitioners [20,44]. More than 70% of the participants in this study incorrectly believed that the fetus absorbs the mother’s calcium for its development, and this leads to dental caries in pregnancy. General practitioners had the same concept about decay development in pregnancy [44]. Also, only 40% of the midwifery students in ours study knew that the second semester is the best timing for receiving dental treatments similar to other studies [31,32]. In contrast, between 80% to 94% of the obstetricians and gynecologists had selected second semester [33,43,44]. 

Lack of knowledge on oral health in pregnancy and at the same time positive attitudes and eagerness of midwives and nurses towards learning, emphasizes design and implementation of educational interventions on oral health [31,32,37,38]. George at al., 2014 designed and implemented a pilot online intervention on midwives in Australia which was successful [25]. In another study midwives in Australia completed a Midwifery Initiated Oral Health online program, and the findings identified that antenatal health care professionals could effectively improve oral health promotion among pregnant women [26]. Both studies were conducted in a non-academic setting. In another study the attitudes and practice of midwifery students on their own oral health were targeted [31]. 

The midwifery students in our study were followed-up three months after the intervention and their knowledge scores were still high. However, three months is not long enough. Several researchers in education believe that repeating the educational material in short intervals or in another word spaced practice is a cost-effective way to advance the effectiveness and efficiency of learning new material [45,46]. This suggestion is in accordance with another study on including content on oral health in pregnancy to the curriculum [47]. For future research examining the effects of periodical oral health care educational sessions on midwifery students’ knowledge and attitudes is highly recommended. 

In this study the current status of midwifery students, their strengths and weaknesses and possible threats to implementation of oral health programs were examined. The midwifery students had no prior exposure to content on oral health in pregnancy, so the responses to attitude questions were interpreted with this pre-assumption in mind. This study has a few limitations. Use of self-administered questionnaires and self-rating of knowledge among midwifery students might raise the risk of self-overestimation or social-desirability [48]. The generalizability of the results to other midwifery schools is limited since the participants are from one school and the size of the case and control groups are small. The reliability score of 0.7 due to small sample size is acceptable. The study had a true experimental design which adds to the value of the study. Therefore, despite limited external generalizability, the internal validity of the results was high. 

Although there are a few interventional studies among midwives, the contribution of our study is that the content was designed to improve knowledge on different areas of gingival health, dental decay, and oral health care in pregnancy in an academic setting [25,31]. The results of this study provide evidence on successful implementation of an educational intervention and highlight the eagerness and interest of future midwives. According to our results there is a significant relationship between attitude and willingness on providing oral health care for pregnant women. Pregnant women in a qualitative study in Iran also highlighted that positive attitude and friendly behavior of the dental health care providers influenced their oral health performance [49]. Literature shows that low attitudes and incorrect beliefs of midwives and nurses can act as a barrier to translating knowledge into practice [50,51]. This indicates that educational interventions besides increasing the knowledge level should also improve and emphasize the attitudes and willingness of the participating health care providers [52,53]. 

Midwives are the main health care providers in contact with pregnant women in family health planning sectors located in various governmental health centers in Iran [21]. Routine care and preventive screening procedures are considered one of the major roles of midwives around the world [54]. At the same time our findings support the idea that midwives can assist expecting mothers in their oral health care. There is low rate of dental visits reported by pregnant women in Iran, United Kingdom and Australia [14,15,16,17]. Therefore, educating midwives on oral health care in pregnancy can be highly beneficial in improving oral health in pregnancy. 

However, in reality and practice incorporating oral health care in the midwifery educational curriculum is a multi-layered process and requires facilitated cooperation of stakeholders, including midwifery faculties and students, course committee planners and educational provosts [55]. Considering the high volume of course work in the midwifery school it took us a considerable amount of time and effort to plan the workshops in this study despite assistance from the school dean and administrative personnel. In addition to administrative obstacles, there is also uncertainty to what extent students will be able to transfer the knowledge gained in educational programs to their practice. Other major limitation factors to consider are insufficient knowledge of pregnant women and midwives, high cost of dental treatments, time restrictions of both patient and providers, dentists’ reluctance in providing treatment to pregnant women, cultural beliefs in pregnant women on oral health and lack of interprofessional collaboration among providers [49,56]. In future we aim to conduct a qualitative study among midwives practicing in family planning sections within public health centers. Their opinions, views and suggested strategies can be used to design and implement a routine sustainable preventive oral health care plan for pregnant women. 

## 5. Conclusions

The knowledge and attitudes of midwifery students in this study on gingival health, dental decay and oral health care were moderately low. However, measurement of students’ knowledge and attitude scores immediately and three months after the workshop demonstrated interesting improvements.

## Figures and Tables

**Table 1:** Questionnaire scales, items within scales, response formats and scoring range for each scale.

Sections	Number of Items	Example	Response Format	Scale Range
1. Perceived Knowledge	3	How do you perceive your understanding on gingival health in pregnant women?	5 options: No knowledge (1) To Extremely high knowledge (5)	3–15
2. Actual Knowledge Gingival health Dental caries Dental and oral health care in pregnancy	3	Which one is not a characteristic of gingival inflammation (gingivitis)? Which teeth are the most prone to dental caries? Which antibiotic is safe for prescribing for pregnant woman?	5 options MCQ ^1^: One best response	0–10
	3			
	4			
3. Attitudes on oral health care in pregnant women	7	I need to learn the skills for educating and monitoring pregnant women for oral health.	5 options: Strongly disagree (1)To strongly agree (5)	7–35

^1^ Multiple Choice Questions.

**Table 2:** Mean and standard deviation of the students’ total knowledge responses in intervention and control groups at pre-test, post-test and 3 months follow-up (Mean and Standard Deviation).

Total Knowledge	Intervention Group	Control Group	Scale Range
	Mean ± SE	Mean ± SE	Min-Max
Pre-test	4.63 ± 0.25	4.79 ± 0.31	0–10
Post-test	9.77 ± 0.09	5.25 ± 0.28	0–10
Follow-up 3 months	8.87 ± 0.15	5.57 ± 0.29	0–10

**Table 3:** Correlation among total attitudes and actual knowledge of midwifery students in the study and their interest and eagerness to attend workshop and provide oral health care in pregnancy.

Domains	Time Interval	Total Attitudes		Total Actual Knowledge	
		Intervention	Control	Intervention	Control
Interest to attend workshop	Pre-test	*r* = 0.57 ^1^	*r* = −0.01	*r* = 0.05	*r* = −0.28
Eagerness to provide oral health care in pregnancy		*r* = 0.48 ^1^	*r* = 0.01	*r* = 0.1	*r* = −0.3
Interest to attend workshop	Post-test	*r* = 0.43 ^2^	*r* = 0.1	*r* = −0.36 ^2^	*r* = −0.27
Eagerness to provide oral health care in pregnancy		*r* = 0.3	*r* = 0.12	*r* = −0.31	*r* = −0.28
Interest to attend workshop	Follow-up 3 Months	*r* = 0.15	*r* = 0.28	*r* = −0.48 ^1^	*r* = −0.32
Eagerness to provide oral health care in pregnancy		*r* = 0.19	*r* = 0.3	*r* = −0.36	*r* = −0.3

^1^*p* < 0.05; ^2^ *p* < 0.01.
